# Supplementary material for: Inactivation of ackA and pta Genes Reduces GlpT Expression and Susceptibility to Fosfomycin in Escherichia coli
Source: Microbiol Spectr. 2023 May 18;11(3):e05069-22. doi: 10.1128/spectrum.05069-22 (PMC10269713; doi:10.1128/spectrum.05069-22)
Supplement: Supplemental file 1 — Supplemental material. Download spectrum.05069-22-s0001.doc, DOC file, 0.1 MB [file spectrum.05069-22-s0001.doc]

**Table S1. MICs of other antimicrobial agents**

| Antimicrobials | CFT073 | CFT073ΔackA | CFT073Δpta |
| --- | --- | --- | --- |
| Levofloxacin  Amikacin  Tetracycline  Piperacillin  Cefotaxime  Aztreonam  Meropenem  Chloramphenicol  Colistin | 0.03  8  2  1  0.06  0.12  0.03  8  2 | 0.03  8  2  1  0.06  0.12  0.03  8  2 | 0.03  8  2  1  0.06  0.12  0.03  8  2 |

Values in the columns are MICs (mg/L).

**Table S2. Strains and plasmids used in this study**

| Strain or plasmid | Relevant genotype/phenotype | Reference |
| --- | --- | --- |
| Strains |  |  |
| CFT073 | Parent strain (ATCC 700928) | ATCC 700928 |
| CFT073ΔackA | *ackA* mutant from CFT073 | This work |
| CFT073Δpta | *pta* mutant from CFT073 | This work |
| CFT073ΔackAΔpta | *ackA* and *pta* double mutant from CFT073 | This work |
| CFT073ΔglpT | *glpT* mutant from CFT073 | This work |
| CFT073ΔackAΔglpT | *ackA* and *glpT* mutant from CFT073 | This work |
| CFT073ΔptaΔglpT | *pta* and *glpT* mutant from CFT073 | This work |
| CFT073ΔuhpT | *uhpT* mutant from CFT073 | This work |
| CFT073ΔackAΔuhpT | *ackA* and *uhpT* mutant from CFT073 | This work |
| CFT073ΔptaΔuhpT | *pta* and *uhpT* mutant from CFT073 | This work |
| CFT073ΔglpR | *glpR* mutant from CFT073 | This work |
| CFT073ΔackAΔglpR | *ackA* and *glpR* mutant from CFT073 | This work |
| CFT073ΔptaΔglpR | *pta* and *glpR* mutant from CFT073 | This work |
| CFT073ΔcpxAR | *cpxAR* mutant from CFT073 | This work |
| CFT073ΔackAΔcpxAR | *ackA* and *cpxAR* mutant from CFT073 | This work |
| CFT073ΔptaΔcpxAR | *pta* and *cpxAR* mutant from CFT073 | This work |
| CFT073Δfis | *fis* mutant from CFT073 | This work |

**Table S2. Strains and plasmids used in this study -*continued***

| Strain or plasmid | Relevant genotype/phenotype | Reference |
| --- | --- | --- |
| Strains |  |  |
| Rosetta(DE3) | T7 expression strain; Cm^R^ | Novagen/EMD Bioscience |
| O157 | EHEC O157:H7 Sakai (RIMD 0509952) | RIMD 0509952 |
| O157ΔackA Δpta | *ackA* and *pta* double mutant from O157 | This work |
| GU2019-E4 | UPEC ESBL producer | This work |
| GU2019-E4ΔackA Δpta | *ackA* and *pta* double mutant from GU2019-E4 | This work |
| Plasmids |  |  |
| pKO3 | Temperature sensitive vector for gene targetting, *sacB*, Cm^R^ | (53) |
| pTrc99K | Vector for IPTG-inducible expression; Km^R^ | (54) |
| pTrc99KackA | *ackA* expression plasmid; Km^R^ | This work |
| pTrc99Kpta | *pta* expression plasmid; Km^R^ | This work |
| pTrc99Kfis | *fis* expression plasmid; Km^R^ | This work |
| pNNglpT-P | *glpT* promoter reporter, Cmr^R^ | (25) |
| pQE80L | Vector for expression of His-tagged protein; Ap^R^ | Qiagen |
| pQE80fis | N-terminal His_6_-Fis overexpression plasmid; Ap^R^ | This work |

Cm^R^ : Chloramphenicol resistance, Km^R^ : Kanamycin resistance, Ap^R^ : Ampicillin resistance

**Table S3. Primers used in this study**

| Primer | DNA sequence (5’ – 3’) | Use |
| --- | --- | --- |
| ackA-delta1 | gcgggatcccgatcggcggcataaaacg | *ackA* mutant construction |
| ackA-delta2 | gtgtgaaatcaggcagtcagcttactcgacatggaagtacc | *ackA* mutant construction |
| ackA -delta3 | ggtacttccatgtcgagtaagctgactgcctgatttcacac | *ackA* mutant construction |
| ackA -delta4 | gcggtcgacgccctgagacataacgaagac | *ackA* mutant construction |
| pta-delta1 | gcgggatcctgctgggtctgaccgaagtg | *pta* mutant construction |
| pta-delta2 | tgatgacgagattactgctgctgacgggacacggtttatcctc | *pta* mutant construction |
| pta-delta3 | aaagaggataaaccgtgtcccgtcagcagcagtaatctcgtc | *pta* mutant construction |
| pta-delta4 | gcggtcgacgttcatcgcccgtcgtgaac | *pta* mutant construction |
| ackApta-delta2 | cggatgatgacgagattactgctgcttactcgacatggaagtacc | *ackA/pta* double mutant construction |
| ackApta-delta3 | ggtacttccatgtcgagtaagcagcagtaatctcgtcatcatc | *ackA/pta* double mutant construction |
| glpT-delta1 | gcgggatccagcgcgcaccgctgtgcag | *glpT* mutant construction |
| glpT-delta2 | tcatgccattagcctccgttgcgaatactcaacattgaaagcc | *glpT* mutant construction |
| glpT-delta3 | cggaggctttcaatgttgagtattcgcaacggaggctaatggc | *glpT* mutant construction |
| glpT-delta4 | gcggtcgaccttcaaaggtgtgcacccgg | *glpT* mutant construction |
| uhpT-delta1 | gcgggatcctgtggctgatgccatttgc | *uhpT* mutant construction |
| uhpT-delta2 | cagttacgtttatgccactgtcaagaaagccagcatgggttac | *uhpT* mutant construction |
| uhpT-delta3 | aggagtaacccatgctggctttcttgacagtggcataaacgtaac | *uhpT* mutant construction |
| uhpT-delta4 | gcggtcgacttgctcggcggctttggtc | *uhpT* mutant construction |
| cpxAR-delta1 | gcgggatccctgcctgtgcgcgcacagc | *cpxAR* mutant construction |
| cpxAR-delta2 | ctacaaatgcggagtttaactccgtaacaggattttattcattg | *cpxAR* mutant construction |
| cpxAR-delta3 | taaacaatgaataaaatcctgttacggagttaaactccgcatttg | *cpxAR mutant construction* |

**Table S3. Primers used in this study -*continued***

| Primer | DNA sequence (5’ – 3’) | Use |
| --- | --- | --- |
| cpxAR-delta4 | gcggtcgacaattcaggtcagccagccgc | *cpxAR* mutant construction |
| glpR-delta1 | gcgggatccctgaagtttgagttctggcg | *glpR* mutant construction |
| glpR-delta2 | gaagcaatgcaggatcagcacagttgtttcattgataaatccctg | *glpR* mutant construction |
| glpR-delta3 | ccagggatttatcaatgaaacaactgtgctgatcctgcattgc | *glpR* mutant construction |
| glpR-delta4 | gcggtcgacaagtggggcgccacgcctc | *glpR* mutant construction |
| fis-delta1 | gcgggatccctgagtacgacagtattcgg | *fis* mutant construction |
| fis-delta2 | ttagctaacctgaattagttcatttcgaacatagttctgtcag | *fis* mutant construction |
| fis-delta3 | gagctgacagaactatgttcgaaatgaactaattcaggttagc | *fis* mutant construction |
| fis-delta4 | gcggtcgacgttttttcgcctgcactcc | *fis* mutant construction |
| pTrcackA-F  pTrcackA-R  pTrcpta-F  pTrcpta-R  pTrcfis-F  pTrcfis-R  pQE80fis-F  pQE80fis-R  glpT-PF  glpT-PR  rhlR-PF  rhlR-PR | gcgccatggcgagtaagttagtactg  gcgggatcctcaggcagtcaggcggctc  gcgccatggtgtcccgtattattatgctg  gcgagatctttactgctgctgtgcagactg  gcgccatggtcgaacaacgcgtaaattc  gcgggatccttagttcatgccgtattttttc  gcgggatccttcgaacaacgcgtaaattctg  gcgaagcttagttcatgccgtattttttc  gcggcggccgctcacttgattgcgagtcgcg  gcgaagctttgaaagcctccgtggcccgtg  gcgggatccgaccaagtccccgtgtcgtg  gcgggatcctcgccatcatcctgagcatc | pTrc99KackA construction  pTrc99KackA construction  pTrc99Kpta construction  pTrc99Kpta construction  pTrc99Kfis construction  pTrc99Kfis construction  pQE80fis construction  pQE80fis construction  Probe preparation for gel shift assay  Probe preparation for gel shift assay  Probe preparation for gel shift assay  Probe preparation for gel shift assay |

**Table S3. Primers used in this study -*continued***

| Primer | DNA sequence (5’ – 3’) | Use |
| --- | --- | --- |
| rrsA-qPCR-F  rrsA-qPCR-R  rpoD-qPCR-F  rpoD-qPCR-R glpT-qPCR-F  glpT-qPCR-R  uhpT-qPCR-F  uhpT-qPCR-R  murA-qPCR-F  murA-qPCR-R  crp-qPCR-F  crp-qPCR-R  glpR-qPCR-F  glpR-qPCR-R  cpxP-qPCR-F  cpxP-qPCR-R  torC-qPCR-F  torC-qPCR-R  fnr-qPCR-F  fnr-qPCR-R | cggtggagcatgtggtttaa  gaaaacttccgtggatgtcaaga  caagccgtggtcggaaaa  gggcgcgatgcacttct  tgcccgcaggtttgattc  ccatggcacaaagcccata  aagccgaccctggacctt  acggtttgaaccacattttgc  cacaatttccggcgctaaa  gccagtagagcggcaaaaag  ccgtcaggaaatcggtcaga  tgcgtcccacggtttca  gccacccagaccgaagaaa  ttgctccgccactttgc  tggagacaatgcatcgtcttg  gcgcgcacagcgtttt  ttgccgagcgtgaatgg  gcgacaggttgccgagtt  aggaacgtctggctgcattc  gccgcgttgggcaaa | Quantitative real-time PCR  Quantitative real-time PCR  Quantitative real-time PCR  Quantitative real-time PCR  Quantitative real-time PCR  Quantitative real-time PCR  Quantitative real-time PCR  Quantitative real-time PCR  Quantitative real-time PCR  Quantitative real-time PCR  Quantitative real-time PCR  Quantitative real-time PCR  Quantitative real-time PCR  Quantitative real-time PCR  Quantitative real-time PCR  Quantitative real-time PCR  Quantitative real-time PCR  Quantitative real-time PCR  Quantitative real-time PCR  Quantitative real-time PCR |

**Table S3. Primers used in this study -*continued***

| Primer | DNA sequence (5’ – 3’) | Use |
| --- | --- | --- |
| fis-qPCR-F  fis-qPCR-R  ihfA-qPCR-F  ihfA-qPCR-R  ihfB-qPCR-F  ihfB-qPCR-R  plaR-qPCR-F  plaR-qPCR-R | ccctgcgtgactcggttaa  cctgaccattcagttgagcaaa  cgggacgtaacccgaaaa  cacgcgccgtgctgta  agcatatggcctcgactcttg  ccgaaaccgcggatttc  gggaaagccatcagcatga  gcagctcggtaatggtattgc | Quantitative real-time PCR  Quantitative real-time PCR  Quantitative real-time PCR  Quantitative real-time PCR  Quantitative real-time PCR  Quantitative real-time PCR  Quantitative real-time PCR  Quantitative real-time PCR |

**Supplemental figure legends**

**Figure S1. Comparison of DNA sequences of *ackA-pta* and its upstream region.** The *ackA* and *pta* genes start at positions 338 and 1615, and end at positions 1540 and 3759, respectively. DNA sequences of CFT073 and EHEC O157:H7 Sakai strains were obtained from the GenBank (Accession numbers are CP051263 and NC_002695, respectively). Bases that differ from the other two strains were marked in black.

**Figure S2. Comparison of DNA sequences of *glpT* and its upstream region.** The *glpT* gene starts at a position 273, and end at a position 1631. DNA sequences of CFT073 and EHEC O157:H7 Sakai strains were obtained from the GenBank (Accession numbers are CP051263 and NC_002695, respectively). Bases that differ from the other two strains were marked in black.

**Figure S3. Comparison of DNA sequences of *fis* and its upstream region.** The *fis* gene starts at a position 301, and end at a position 597. DNA sequences of CFT073 and EHEC O157:H7 Sakai strains were obtained from the GenBank (Accession numbers are CP051263 and NC_002695, respectively). Bases that differ from the other two strains were marked in black.
