# Supplementary material for: Inactivation of ackA and pta Genes Reduces GlpT Expression and Susceptibility to Fosfomycin in Escherichia coli
Source: Microbiol Spectr. 2023 May 18;11(3):e05069-22. doi: 10.1128/spectrum.05069-22 (PMC10269713; doi:10.1128/spectrum.05069-22)
Supplement: Supplemental file 2 — Supplemental material. Download spectrum.05069-22-s0002.pdf, PDF file, 1.5 MB [file spectrum.05069-22-s0002.pdf]

```
GU2019-E4_ackA     1 GCTTCACCTCAACTTCACATATAAAGATTCAAAAATTTGTGCAAATTCACAACTCAGCGGGACAACGTTCAAAACATTTTGTCTTCCATACCCACTATCA 100
CFT073_ackA        1 GCTTCACCTCAACTTCACATATAAAGATTCAAAAATTTGTGCAAATTCACAACTCAGCGGGACAACGTTCAAAACATTTTGTCTTCCATACCCACTATCA 100
O157Sakai ackA     1 GCTTCACCTCAACTTCACATATAAAGATTCAAAAATTTGTGCAAATTCACAACTCAGCGGGACAACGTTCAAAACATTTTGTCTTCCATACCCACTATCA 100

GU2019-E4_ackA   101 GGTATCCTTTAGCAGCCTGAAGGCCTAAGTAGTACATATTCATTGAGTCGTCAAATTCATATACATTATGCCATTGGCTGAAAATTACGCAAAATGGCAT 200
CFT073_ackA      101 GGTATCCTTTAGCAGCCTGAAGGCCTAAGTAGTACATATTCATTGAGTCGTCAAATTCATATACATTATGCCATTGGCTGAAAATTACGCAAAATGGCAT 200
O157Sakai ackA   101 GGTATCCTTTAGCAGCCTGAAGGCCTAAGTAGTACATATTCATTGAGTCGTCAAATTCATATACATTATGCCATTGGCTGAAAATTACGCAAAATGGCAT 200

GU2019-E4_ackA   201 AGACTCAAGATATTTCTTCCATCATGCAAAAAAAATTTGCAGTGCATGATGTTAATCATAAATGTCGGTGTCATCATGCGCTACGCTCTATGGCTCCCTG 300
CFT073_ackA      201 AGACTCAAGATATTTCTTCCATCATGCAAAAAAAATTTGCAGTGCATGATGTTAATCATAAATGTCGGTGTCATCATGCGCTACGCTCTATGGCTCCCTG 300
O157Sakai ackA   201 AGACTCAAGATATTTCTTCCATCATGCAAAAAAAATTTGCAGTGCATGATGTTAATCATAAATGTCGGTGTCATCATGCGCTACGCTCTATGGCTCCCTG 300

GU2019-E4_ackA   301 ACGTTTTTTTAGCCACGTATCAATTATAGGTACTTCCATGTCGAGTAAGTTAGTACTGGTTCTGAACTGCGGTAGTTCTTCACTGAAATTTGCCATCATC 400
CFT073_ackA      301 ACGTTTTTTTAGCCACGTATCAATTATAGGTACTTCCATGTCGAGTAAGTTAGTACTGGTTCTGAACTGCGGTAGTTCTTCACTGAAATTTGCCATCATC 400
O157Sakai ackA   301 ACGTTTTTTTAGCCACGTATCAATTATAGGTACTTCCATGTCGAGTAAGTTAGTACTGGTTCTGAACTGCGGTAGTTCTTCACTGAAATTTGCCATCATC 400

GU2019-E4_ackA   401 GATGCAGTAAATGGTGAAGAGTACCTTTCTGGTTTAGCCGAATGTTTCCACCTGCCCGAAGCACGTATCAAATGGAAAATGGACGGCAATAAACAGGAAG 500
CFT073_ackA      401 GATGCAGTAAATGGTGAAGAGTACCTTTCTGGTTTAGCCGAATGTTTCCACCTGCCCGAAGCACGTATCAAATGGAAAATGGACGGCAATAAACAGGAAG 500
O157Sakai ackA   401 GATGCAGTAAATGGTGAAGAGTACCTTTCTGGTTTAGCCGAATGTTTCCACCTGCCCGAAGCACGTATCAAATGGAAAATGGACGGCAATAAACAGGAAG 500

GU2019-E4_ackA   501 CGGCTTTAGGTGCAGGCGCCGCTCACAGCGAAGCGCTCAACTTTATCGTTAATACTATTCTGGCACAAAAACCAGAACTGTCTGCGCAGCTGACTGCTAT 600
CFT073_ackA      501 CGGCTTTAGGTGCAGGCGCCGCTCACAGCGAAGCGCTCAACTTTATCGTTAATACTATTCTGGCACAAAAACCAGAACTGTCTGCGCAGCTGACTGCTAT 600
O157Sakai ackA   501 CGGCTTTAGGTGCAGGCGCCGCTCACAGCGAAGCGCTCAACTTTATCGTTAATACTATTCTGGCACAAAAACCAGAACTGTCTGCGCAGCTGACTGCTAT 600

GU2019-E4_ackA   601 CGGTCACCGTATCGTACACGGCGGCGAAAAGTATACCAGCTCCGTAGTGATTGATGAGTCTGTTATTCAGGGTATCAAAGATGCAGCTTCTTTTGCACCG 700
CFT073_ackA      601 CGGTCACCGTATCGTACACGGCGGCGAAAAGTATACCAGCTCCGTAGTGATCGATGAGTCTGTTATTCAGGGTATCAAAGATGCAGCTTCTTTTGCACCG 700
O157Sakai ackA   601 CGGTCACCGTATCGTACACGGCGGCGAAAAGTATACCAGCTCCGTAGTGATCGATGAGTCTGTTATTCAGGGTATCAAAGATGCAGCTTCTTTTGCACCG 700

GU2019-E4_ackA   701 CTGCACAACCCGGCTCACCTGATCGGTATCGAAGAAGCTCTGAAATCTTTCCCACAGCTGAAAGACAAAAACGTTGCTGTATTTGACACCGCATTCCACC 800
CFT073_ackA      701 CTGCACAACCCGGCTCACCTGATCGGTATCGAAGAAGCTCTGAAATCTTTCCCACAGCTGAAAGACAAAAACGTTGCTGTATTTGACACCGCGTTCCACC 800
O157Sakai ackA   701 CTGCACAACCCGGCTCACCTGATCGGTATCGAAGAAGCTCTGAAATCTTTCCCACAGCTGAAAGACAAAAACGTTGCTGTATTTGACACCGCGTTCCACC 800

GU2019-E4_ackA   801 AGACTATGCCGGAAGAGTCTTATCTCTACGCCCTGCCGTACAACCTGTACAAAGAGCACGGCATCCGTCGTTACGGCGCGCACGGCACCAGCCACTTCTA 900
CFT073_ackA      801 AGACTATGCCGGAAGAGTCTTACCTCTACGCCCTGCCGTACAACCTGTACAAAGAGCACGGCATCCGTCGTTACGGCGCGCACGGCACCAGCCACTTCTA 900
O157Sakai ackA   801 AGACTATGCCGGAAGAGTCTTACCTCTACGCCCTGCCGTACAACCTGTACAAAGAGCACGGCATCCGTCGTTACGGCGCGCACGGCACCAGCCACTTCTA 900

GU2019-E4_ackA   901 TGTAACCCAGGAAGCGGCAAAAATGCTGAACAAACCGGTAGAAGAACTGAACATCATCACCTGCCACCTGGGCAACGGTGGTTCCGTTTCTGCTATCCGC 1000
CFT073_ackA      901 TGTAACCCAGGAAGCGGCAAAAATGCTGAACAAACCGGTAGAAGAACTGAACATCATCACCTGCCACCTGGGCAACGGTGGTTCCGTTTCTGCTATCCGC 1000
O157Sakai ackA   901 TGTAACCCAGGAAGCGGCAAAAATGCTGAACAAACCGGTAGAAGAACTGAACATCATCACCTGCCACCTGGGCAACGGTGGTTCCGTTTCTGCTATCCGC 1000

GU2019-E4_ackA  1001 AACGGTAAATGCGTTGACACCTCTATGGGCCTGACCCCGCTGGAAGGTCTGGTCATGGGTACCCGTTCTGGTGATATCGATCCGGCGATCATCTTCCACC 1100
CFT073_ackA     1001 AACGGTAAATGCGTTGACACCTCTATGGGCCTGACCCCGCTGGAAGGTCTGGTCATGGGTACCCGTTCTGGTGATATCGATCCGGCGATCATCTTCCACC 1100
O157Sakai ackA  1001 AACGGTAAATGCGTTGACACCTCTATGGGCCTGACCCCGCTGGAAGGTCTGGTCATGGGTACCCGTTCTGGTGATATCGATCCGGCGATCATCTTCCACC 1100

GU2019-E4_ackA  1101 TGCACGACACCCTGGGCATGAGCGTTGACGCAATCAACAAACTGCTGACCAAAGAGTCTGGCCTGCTGGGTCTGACCGAAGTGACCAGCGACTGCCGCTA 1200
CFT073_ackA     1101 TGCACGACACCCTGGGCATGAGCGTTGACGCAATCAACAAACTGCTAACCAAAGAGTCTGGCCTGCTGGGTCTGACCGAAGTGACCAGCGACTGCCGCTA 1200
O157Sakai ackA  1101 TGCACGACACCCTGGGCATGAGCGTTGACGCAATCAACAAACTGCTGACCAAAGAGTCTGGCCTGCTGGGTCTGACCGAAGTGACCAGCGACTGCCGCTA 1200

GU2019-E4_ackA  1201 TGTTGAAGACAACTACGCGACGAAAGAAGACGCGAAGCGCGCAATGGACGTTTACTGCCACCGCCTGGCCAAATACATCGGTGCCTACACTGCGCTGATG 1300
CFT073_ackA     1201 TGTTGAAGACAACTACGCGACGAAAGAAGACGCGAAGCGCGCAATGGACGTTTACTGCCACCGCCTGGCCAAATACATCGGTGCCTACACTGCGCTGATG 1300
O157Sakai ackA  1201 TGTTGAAGACAACTACGCGACGAAAGAAGACGCGAAGCGCGCAATGGACGTTTACTGCCACCGCCTGGCGAAATACATCGGTGCCTACACTGCGCTGATG 1300

GU2019-E4_ackA  1301 GATGGTCGTCTGGACGCTGTTGTATTCACCGGTGGTATCGGTGAAAATGCCGCGATGGTTCGTGAACTGTCTCTGGGCAAACTGGGCGTGCTGGGCTTTG 1400
CFT073_ackA     1301 GATGGTCGTCTGGACGCTGTTGTATTCACCGGTGGTATCGGTGAAAATGCCGCGATGGTTCGTGAACTGTCTCTGGGCAAACTGGGCGTGCTGGGCTTTG 1400
O157Sakai ackA  1301 GATGGTCGTCTGGACGCTGTTGTATTCACCGGTGGTATCGGTGAAAATGCCGCGATGGTCCGTGAACTGTCTCTGGGCAAACTGGGCGTGCTGGGCTTTG 1400

GU2019-E4_ackA  1401 AAGTTGATCATGAACGCAACCTGGCTGCACGTTTCGGCAAATCTGGTTTCATCAACAAAGAAGGTACCCGTCCTGCGGTGGTTATCCCAACCAACGAAGA 1500
CFT073_ackA     1401 AAGTTGATCATGAACGCAACCTGGCTGCACGTTTCGGCAAATCTGGTTTCATCAACAAAGAAGGTACCCGTCCTGCGGTGGTTATCCCAACCAACGAAGA 1500
O157Sakai ackA  1401 AAGTTGATCATGAACGCAATCTGGCTGCACGTTTCGGCAAATCTGGTTTCATCAACAAAGAAGGTACCCGTCCTGCGGTGGTTATCCCAACCAACGAAGA 1500

GU2019-E4_ackA  1501 ACTGGTTATCGCGCAAGACGCGAGCCGCCTGACTGCCTGATTTCACACCGCCAGCTCAGCTGGCGGTGCTGTTTTGTAACCCGCCAAATCGGCGGTAACG 1600
CFT073_ackA     1501 ACTGGTTATCGCGCAAGACGCGAGCCGCCTGACTGCCTGATTTCACACCGCCAGCTCAGCTGGCGGTGCTGTTTTGTAACCCGCCAAATCGGCGGTAACG 1600
O157Sakai ackA  1501 ACTGGTTATCGCGCAAGACGCGAGCCGCCTGACTGCCTGATTTCACACCGCCAGCTCAGCTGGCGGTGCTGTTTTGTAACCCGCCAAATCGGCGGTAACG 1600

GU2019-E4_ackA  1601 AAAGAGGATAAACCGTGTCCCGTATTATTATGCTGATCCCTACCGGAACCAGCGTCGGTCTGACCAGCGTCAGCCTTGGCGTGATCCGTGCAATGGAACG 1700
CFT073_ackA     1601 AAAGAGGATAAACCGTGTCCCGTATTATTATGCTGATCCCTACCGGAACCAGCGTCGGTCTGACCAGCGTCAGCCTTGGCGTGATCCGTGCAATGGAACG 1700
O157Sakai ackA  1601 AAAGAGGATAAACCGTGTCCCGTATTATTATGCTGATCCCTACCGGAACCAGCGTCGGTCTGACCAGCGTCAGCCTTGGCGTGATCCGTGCAATGGAACG 1700

GU2019-E4_ackA  1701 CAAAGGCGTTCGTCTGAGCGTTTTCAAACCTATCGCTCAGCCGCGTACCGGTGGCGATGCGCCCGATCAGACTACGACTATCGTGCGTGCGAACTCTTCC 1800
CFT073_ackA     1701 CAAAGGCGTTCGTCTGAGCGTTTTCAAACCTATCGCTCAGCCGCGTACCGGTGGCGATGCGCCCGATCAGACTACGACTATCGTGCGTGCGAACTCTTCC 1800
O157Sakai ackA  1701 CAAAGGCGTTCGTCTGAGCGTTTTCAAACCTATCGCTCAGCCGCGTACCGGTGGCGATGCGCCCGATCAGACTACGACTATCGTGCGTGCGAACTCTTCC 1800

GU2019-E4_ackA  1801 ACCACGACGGCCGCTGAACCGCTGAAAATGAGCTACGTTGAAGGTCTGCTTTCCAGCAATCAGAAAGATGTGCTGATGGAAGAGATCATCGCGAACTACC 1900
CFT073_ackA     1801 ACCACGACGGCCGCTGAACCGCTGAAAATGAGCTACGTTGAAGGTCTGCTTTCCAGCAATCAGAAAGATGTGCTGATGGAAGAGATCATCGCGAACTACC 1900
O157Sakai ackA  1801 ACCACGACGGCCGCTGAACCGCTGAAAATGAGCTACGTTGAAGGTCTGCTTTCCAGCAATCAGAAAGATGTGCTGATGGAAGAGATCGTCGCAAACTACC 1900

GU2019-E4_ackA  1901 ACGCTAACACCAAAGACGCTGAAGTCGTTCTGGTGGAAGGTCTGGTCCCGACACGTAAGCACCAGTTTGCCCAGTCTCTGAACTACGAAATCGCCAAAAC 2000
CFT073_ackA     1901 ACGCTAACACCAAAGACGCTGAAGTCGTTCTGGTGGAAGGTCTGGTCCCGACACGTAAGCACCAGTTTGCCCAGTCTCTGAACTACGAAATCGCCAAAAC 2000
O157Sakai ackA  1901 ACGCTAACACCAAAGACGCTGAAGTCGTTCTGGTTGAAGGTCTGGTCCCGACACGTAAGCACCAATTTGCCCAGTCTCTGAACTACGAAATCGCTAAAAC 2000

GU2019-E4_ackA  2001 GCTGAACGCAGAAATCGTCTTCGTTATGTCTCAGGGCACTGATACTCCGGAACAGTTGAAAGAGCGTATCGAACTGACTCGCAACAGCTTCGGCGGTGCA 2100
CFT073_ackA     2001 GCTGAACGCAGAAATCGTCTTCGTTATGTCTCAGGGCACTGATACTCCGGAACAGTTGAAAGAGCGTATCGAACTGACTCGCAACAGCTTCGGCGGTGCA 2100
O157Sakai ackA  2001 GCTGAATGCGGAAATCGTCTTCGTTATGTCTCAGGGCACTGACACCCCGGAACAGCTGAAAGAGCGTATCGAACTGACCCGCAACAGCTTCGGCGGTGCC 2100

GU2019-E4_ackA  2101 AAAAACACCAATATTACCGGCGTTATCGTTAACAAACTGAACGCTCCGGTTGATGAGCAGGGTCGTACCCGTCCGGATCTGTCCGAGATTTTTGACGACT 2200
CFT073_ackA     2101 AAAAACACCAATATTACCGGCGTTATCGTTAACAAACTGAACGCTCCGGTTGATGAGCAGGGTCGTACCCGTCCGGATCTGTCCGAGATTTTTGACGACT 2200
O157Sakai ackA  2101 AAAAACACCAACATCACCGGCGTTATCGTTAACAAACTGAACGCACCGGTTGATGAACAGGGTCGTACTCGCCCGGATCTGTCCGAGATTTTCGACGACT 2200

GU2019-E4_ackA  2201 CCACCAAAGCAAAAGTGAACAACGTTGATCCGGCGAAGCTGCAAGAATCCAGCCCGCTGCCGGTTCTCGGCGCTGTGCCGTGGAGCTTTGACCTGATCGC 2300
CFT073_ackA     2201 CCACCAAAGCAAAAGTGAACAACGTTGATCCGGCGAAGCTGCAAGAATCCAGCCCGCTGCCGGTTCTCGGCGCTGTGCCGTGGAGCTTTGACCTGATCGC 2300
O157Sakai ackA  2201 CTTCCAAAGCTAAAGTAAACAATGTTGATCCGGCGAAGCTGCAAGAATCCAGCCCGCTGCCGGTTCTCGGCGCTGTGCCGTGGAGCTTTGACCTGATCGC 2300

GU2019-E4_ackA  2301 GACTCGTGCGATCGATATGGCTCGCCACCTGAATGCGACCATCATCAACGAAGGCGACATCAATACTCGCCGCGTTAAATCCGTCACTTTCTGCGCACGC 2400
CFT073_ackA     2301 GACTCGTGCGATCGATATGGCTCGCCACCTGAATGCGACCATCATCAACGAAGGCGACATCAATACTCGCCGCGTTAAATCCGTCACTTTCTGCGCACGC 2400
O157Sakai ackA  2301 GACTCGTGCGATCGATATGGCTCGCCACCTGAATGCGACCATCATCAACGAAGGCGACATCAATACTCGCCGCGTTAAATCCGTCACTTTCTGCGCACGC 2400

GU2019-E4_ackA  2401 AGCATTCCGCACATGCTGGAGCACTTCCGTGCCGGTTCTCTGCTGGTGACTTCCGCAGACCGCCCTGACGTGCTGGTTGCCGCTTGCCTGGCTGCCATGA 2500
CFT073_ackA     2401 AGCATTCCGCACATGCTGGAGCACTTCCGTGCCGGTTCTCTGCTGGTGACTTCCGCAGACCGCCCTGACGTGCTGGTTGCCGCTTGCCTGGCTGCCATGA 2500
O157Sakai ackA  2401 AGCATTCCGCACATGCTGGAGCACTTCCGTGCCGGTTCTCTGCTGGTGACTTCCGCAGACCGTCCTGACGTGCTGGTTGCCGCTTGCCTGGCTGCCATGA 2500

GU2019-E4_ackA  2501 ACGGCGTAGAAATCGGTGCCCTGCTGCTGACTGGCGGCTACGAAATGGACGCGCGCATTTCTAAACTGTGCGAACGTGCTTTCGCTACCGGCCTGCCGGT 2600
CFT073_ackA     2501 ACGGCGTAGAAATCGGTGCCCTGCTGCTGACTGGCGGCTACGAAATGGACGCGCGCATTTCTAAACTGTGCGAACGTGCTTTCGCTACTGGCCTGCCGGT 2600
O157Sakai ackA  2501 ACGGCGTAGAAATCGGTGCCCTGCTGCTGACTGGCGGCTACGAAATGGACGCGCGCATTTCTAAACTGTGCGAACGTGCTTTCGCTACCGGCCTGCCGGT 2600

GU2019-E4_ackA  2601 ATTTATGGTGAACACCAACACCTGGCAGACTTCTCTGAGCCTGCAGAGCTTCAACCTGGAAGTTCCGGTTGACGATCACGAGCGTATCGAGAAAGTTCAG 2700
CFT073_ackA     2601 ATTTATGGTGAACACCAACACCTGGCAGACTTCTCTTAGCCTGCAGAGCTTCAACCTGGAAGTTCCGGTTGACGATCATGAGCGTATCGAAAAAGTTCAG 2700
O157Sakai ackA  2601 ATTTATGGTGAACACCAACACCTGGCAGACTTCTCTGAGCCTGCAGAGCTTCAACCTGGAAGTTCCAGTTGACGATCACGAGCGTATCGAGAAAGTTCAG 2700

GU2019-E4_ackA  2701 GAATACGTTGCTAACTACATCAACGCTGACTGGATCGATTCTCTGACTGCCACTTCTGAGCGCAGCCGTCGTCTGTCTCCGCCAGCGTTCCGTTATCAGC 2800
CFT073_ackA     2701 GAATACGTGGCTAACTACATCAACGCTGACTGGATCGATTCTCTGACTGCCACTTCTGAGCGCAGCCGTCGTCTGTCTCCGCCAGCGTTCCGTTATCAGC 2800
O157Sakai ackA  2701 GAATACGTTGCTAACTACATCAACGCTGACTGGATCGATTCTCTGACTGCCACTTCTGAGCGCAGCCGTCGTCTGTCTCCGCCAGCGTTCCGTTATCAGC 2800

GU2019-E4_ackA  2801 TGACTGAACTTGCGCGCAAAGCGGGCAAACGTATCGTTCTGCCGGAAGGTGACGAACCGCGTACCGTTAAAGCAGCCGCTATCTGTGCTGAACGTGGTAT 2900
CFT073_ackA     2801 TGACTGAACTTGCGCGCAAAGCGGGCAAACGTATCGTTCTGCCGGAAGGTGACGAACCGCGTACCGTTAAAGCAGCCGCTATCTGTGCTGAACGTGGTAT 2900
O157Sakai ackA  2801 TGACTGAACTTGCGCGCAAAGCGGGCAAACGTATCGTACTGCCGGAAGGTGACGAACCGCGTACCGTTAAAGCAGCCGCTATCTGTGCTGAACGTGGTAT 2900

GU2019-E4_ackA  2901 CGCAACTTGCGTACTGCTGGGTAATCCGGCAGAGATCAACCGTGTTGCAGCCTCTCAGGGTGTAGAACTGGGTGCAGGCATTGAAATCGTTGATCCAGAA 3000
CFT073_ackA     2901 CGCAACTTGCGTACTGCTGGGTAATCCGGCAGAGATCAACCGTGTTGCAGCCTCTCAGGGTGTAGAACTGGGTGCAGGCATTGAAATCGTTGATCCAGAA 3000
O157Sakai ackA  2901 CGCAACTTGCGTACTGCTGGGTAATCCGGCAGAGATCAACCGTGTTGCAGCGTCTCAGGGTGTAGAACTGGGTGCAGGCATTGAAATCGTTGATCCAGAA 3000

GU2019-E4_ackA  3001 GTGGTTCGCGAAAACTATGTTGGTCGTCTGGTCGAACTGCGTAAGAACAAAGGCATGACCGAAACCGTTGCCCGCGAACAGCTGGAAGACAACGTGGTTC 3100
CFT073_ackA     3001 GTGGTTCGCGAAAACTATGTTGGTCGTCTGGTCGAACTGCGTAAGAACAAAGGCATGACCGAAACCGTTGCCCGCGAACAGCTGGAAGACAACGTGGTTC 3100
O157Sakai ackA  3001 GTGGTTCGCGAAAGCTATGTTGGTCGTCTGGTCGAGCTGCGTAAGAACAAAGGCATGACCGAAACCGTTGCCCGCGAACAGCTGGAAGACAACGTGGTGC 3100

GU2019-E4_ackA  3101 TCGGTACGCTGATGCTGGAACAAGATGAAGTTGATGGTCTGGTTTCCGGTGCTGTTCACACCACCGCAAACACCATCCGTCCGCCGCTGCAGCTGATCAA 3200
CFT073_ackA     3101 TCGGTACGCTGATGCTGGAACAAGATGAAGTTGATGGTCTGGTTTCCGGTGCTGTTCACACCACCGCAAACACCATCCGTCCGCCGCTGCAGCTGATCAA 3200
O157Sakai ackA  3101 TCGGTACGCTGATGCTGGAACAGGATGAAGTTGATGGTCTGGTTTCCGGTGCTGTTCACACTACCGCAAACACCATCCGTCCGCCGCTGCAGCTGATCAA 3200

GU2019-E4_ackA  3201 AACTGCACCGGGCAGCTCCCTGGTATCTTCCGTGTTCTTCATGCTGTTGCCGGAACAGGTTTACGTTTACGGTGACTGTGCGATCAACCCGGATCCGACC 3300
CFT073_ackA     3201 AACTGCACCGGGCAGCTCCCTGGTATCTTCCGTGTTCTTCATGCTGTTGCCGGAACAGGTTTACGTTTACGGTGACTGTGCGATCAACCCGGATCCGACC 3300
O157Sakai ackA  3201 AACTGCACCGGGCAGCTCCCTGGTATCTTCCGTGTTCTTCATGCTGCTGCCGGAACAGGTTTACGTTTACGGTGACTGTGCGATCAACCCGGATCCGACC 3300

GU2019-E4_ackA  3301 GCAGAACAGCTGGCAGAAATCGCGATTCAGTCCGCTGATTCCGCTGCGGCCTTCGGTATCGAACCGCGCGTTGCTATGCTCTCCTACTCCACCGGTACTT 3400
CFT073_ackA     3301 GCAGAACAGCTGGCAGAAATCGCGATTCAGTCCGCTGATTCCGCTGCGGCCTTCGGTATCGAACCGCGCGTTGCTATGCTCTCCTACTCCACCGGTACTT 3400
O157Sakai ackA  3301 GCAGAACAGCTGGCAGAAATCGCGATTCAGTCCGCTGATTCCGCTGCAGCCTTCGGTATCGAACCGCGCGTTGCTATGCTCTCCTACTCTACCGGTACTT 3400

GU2019-E4_ackA  3401 CTGGTGCTGGTAGCGACGTAGAAAAAGTTCGCGAAGCAACTCGTCTGGCGCAGGAAAAACGTCCTGATCTGATGATCGACGGTCCGCTGCAGTACGACGC 3500
CFT073_ackA     3401 CTGGTGCTGGTAGCGACGTAGAAAAAGTTCGCGAAGCAACTCGTCTGGCGCAGGAAAAACGTCCTGATCTGATGATCGACGGTCCGCTGCAGTACGACGC 3500
O157Sakai ackA  3401 CTGGTGCAGGTAGCGACGTAGAAAAAGTTCGCGAAGCAACTCGTCTGGCGCAGGAAAAACGTCCTGACCTGATGATCGACGGTCCGCTGCAGTACGACGC 3500

GU2019-E4_ackA  3501 TGCGGTAATGGCTGACGTTGCGAAATCCAAAGCACCGAACTCTCCGGTTGCAGGTCGCGCTACCGTGTTCATCTTCCCGGATCTGAACACCGGTAACACC 3600
CFT073_ackA     3501 TGCGGTAATGGCTGACGTTGCGAAATCCAAAGCACCGAACTCTCCGGTTGCAGGTCGCGCTACCGTGTTCATCTTCCCGGATCTGAACACCGGTAACACC 3600
O157Sakai ackA  3501 TGCGGTAATGGCTGACGTTGCGAAATCCAAAGCGCCGAACTCTCCGGTTGCAGGTCGCGCTACCGTGTTCATCTTCCCGGATCTGAACACCGGTAACACC 3600

GU2019-E4_ackA  3601 ACCTACAAAGCGGTACAGCGTTCTGCTGACCTGATCTCTATCGGACCGATGCTGCAGGGCATGCGCAAGCCGGTTAACGACCTGTCCCGTGGCGCACTGG 3700
CFT073_ackA     3601 ACCTACAAAGCGGTACAGCGTTCTGCTGACCTGATCTCTATCGGACCGATGCTGCAGGGTATGCGCAAGCCGGTTAACGACCTGTCCCGTGGCGCACTGG 3700
O157Sakai ackA  3601 ACCTACAAAGCGGTACAGCGTTCTGCCGACCTGATCTCCATCGGGCCGATGCTGCAGGGTATGCGCAAGCCGGTTAACGACCTGTCCCGTGGCGCACTGG 3700

GU2019-E4_ackA  3701 TTGATGATATCGTCTACACCATCGCGCTGACTGCAATTCAGTCTGCACAGCAGCAGTAA                                          3759
CFT073_ackA     3701 TTGATGATATCGTCTACACCATCGCGCTGACTGCGATTCAGTCTGCACAGCAGCAGTAA                                          3759
O157Sakai ackA  3701 TTGACGATATCGTCTACACCATCGCGCTGACTGCGATTCAGTCTGCACAGCAGCAGTAA                                          3759
```
