# Supplementary material for: Inactivation of ackA and pta Genes Reduces GlpT Expression and Susceptibility to Fosfomycin in Escherichia coli
Source: Microbiol Spectr. 2023 May 18;11(3):e05069-22. doi: 10.1128/spectrum.05069-22 (PMC10269713; doi:10.1128/spectrum.05069-22)
Supplement: Supplemental file 3 — Supplemental material. Download spectrum.05069-22-s0003.pdf, PDF file, 0.7 MB [file spectrum.05069-22-s0003.pdf]

```
GU2019-E4_glpT     1 TGTTATCCCTCAGAAGTTCGTTTTTTACCATTTAGCCATAGTAAAAACATGAATTGTTTGATTTCGCGCATATTCGCTCATAATTCGAAAGTGAAACGTG 100
CFT073_glpT        1 TGTTATCCCTCTGAAGTTCGTTTTTTACCATTTAGCCATAGTAAAAACATGAATTGTTTGATTTCGCGCATATTCGCTCATAATTCGAAAGTGAAACGTG 100
O157Sakai_glpT     1 TGTTATCCCTCTGAAGTTCGTTTTTTACCATTTAGCCATAGTAAAAACATGAGTTGTTTGATTTCGCGCATATTCGCTCATAATTCGAAAGTGAGACGTG 100

GU2019-E4_glpT   101 ATTTCATGCGTCATTCTGAACATTTCAACCATCGTATTTAATAATGTGTGCGGTAATTCACATTTAATTTATGAATGTTTTCTTAACATCGCGGCAACTC 200
CFT073_glpT      101 ATTTCATGCGTCATTCTGAACATTTCAACCATCGTATTTAATAATGTGTGCGGTAATTCACATTTAATTTATGAATGTTTTCTTAACATCGCGGCAACTC 200
O157Sakai_glpT   101 ATTTCATGCGTCATTTTGAACATTTTGTAAATCTTATTTAATAATGTGTGCGGCAATTCACATTTAATTTATGAATGTTTTCTTAACATCGCGGCAACTC 200

GU2019-E4_glpT   201 AAGAAACGGCAGGTTCTCCCACTGAATCAGGCTGTTAATCATAAATAAGACCACGGGCCACGGAGGCTATCAATGTTGAGTATTTTTAAACCAGCGCCAC 300
CFT073_glpT      201 AAGAAACGGCAGGTTCTCCCACGGAATCAGGCTGTTAATCATAAATAAGACCACGGGCCACGGAGGCTATCAATGTTGAGTATTTTTAAACCAGCGCCAC 300
O157Sakai_glpT   201 AAGAAACGGCAGGTTCTCTCACTGAATCAGGCTGTTAATCATAAATAAGACCACGGGCCACGGAGGCTTTCAATGTTGAGTATTTTTAAACCAGCGCCAC 300

GU2019-E4_glpT   301 ACAAAGCGCGCTTACCTGCCGCGGAGATCGATCCGACTTATCGTCGATTGCGCTGGCAAATTTTCCTGGGGATATTCTTTGGCTATGCTGCTTACTATTT 400
CFT073_glpT      301 ACAAAGCGCGCTTACCTGCCGCGGAGATCGATCCGACTTATCGTCGATTGCGCTGGCAAATTTTCCTGGGGATATTCTTTGGCTATGCTGCTTACTATTT 400
O157Sakai_glpT   301 ACAAAGCGCGCTTACCTGCCGCGGAGATCGATCCGACTTATCGTCGATTGCGCTGGCAAATTTTCCTGGGGATATTCTTTGGCTATGCTGCTTACTATTT 400

GU2019-E4_glpT   401 GGTTCGTAAGAACTTTGCGCTCGCTATGCCTTATCTGGTTGAGCAGGGATTCTCACGCGGTGATTTAGGTTTTGCCCTTTCGGGGATCTCGATTGCTTAT 500
CFT073_glpT      401 GGTTCGTAAGAACTTTGCGCTCGCTATGCCTTATCTGGTTGAGCAGGGATTCTCACGCGGTGATTTAGGTTTTGCCCTTTCGGGGATCTCGATTGCTTAT 500
O157Sakai_glpT   401 GGTTCGTAAGAACTTTGCGCTCGCTATGCCTTACCTGGTGGAGCAGGGATTCTCACGCGGTGATTTAGGTTTTGCGCTTTCTGGGATCTCGATCGCCTAT 500

GU2019-E4_glpT   501 GGATTTTCGAAATTCATCATGGGTTCGGTATCGGATCGCTCGAATCCGCGCGTTTTCCTGCCCGCAGGTTTGATTCTGGCGGCGGCAGTGATGTTGTTTA 600
CFT073_glpT      501 GGATTTTCGAAATTCATCATGGGTTCGGTATCGGATCGCTCGAATCCGCGCGTTTTCCTGCCCGCAGGTTTGATTCTGGCGGCGGCAGTGATGTTGTTTA 600
O157Sakai_glpT   501 GGATTTTCGAAATTCATCATGGGTTCGGTATCGGATCGCTCGAATCCGCGCGTTTTCCTGCCCGCAGGTTTGATTCTGGCGGCGGCAGTGATGTTGTTTA 600

GU2019-E4_glpT   601 TGGGCTTTGTGCCATGGGCGACGTCGAGCATTGCGGTGATGTTTGTACTGTTGTTCCTCTGCGGTTGGTTCCAGGGGATGGGGTGGCCGCCGTGTGGTCG 700
CFT073_glpT      601 TGGGCTTTGTGCCATGGGCGACGTCGAGCATTGCGGTGATGTTTGTACTGTTGTTCCTCTGCGGTTGGTTCCAGGGGATGGGGTGGCCGCCGTGTGGTCG 700
O157Sakai_glpT   601 TGGGCTTTGTGCCATGGGCGACGTCGAGCATTGCGGTGATGTTTGTACTGTTGTTCCTCTGCGGTTGGTTCCAGGGGATGGGGTGGCCGCCATGCGGTCG 700

GU2019-E4_glpT   701 TACTATGGTGCACTGGTGGTCGCAGAAAGAACGTGGCGGTATTGTGTCAGTGTGGAACTGTGCGCACAACGTCGGTGGTGGTATTCCGCCGCTGCTGTTC 800
CFT073_glpT      701 TACTATGGTGCACTGGTGGTCGCAGAAAGAACGTGGCGGCATTGTGTCAGTGTGGAACTGTGCGCACAACGTCGGTGGTGGTATTCCGCCGCTGCTGTTC 800
O157Sakai_glpT   701 TACTATGGTGCACTGGTGGTCGCAGAAAGAACGTGGCGGCATTGTGTCAGTGTGGAACTGTGCGCACAACGTCGGTGGTGGTATTCCGCCGCTGCTGTTC 800

GU2019-E4_glpT   801 CTGCTGGGGATGGCCTGGTTCAATGACTGGCACGCGGCGCTCTATATGCCCGCTTTCTGCGCCATTCTGGTGGCACTGTTCGCCTTTGCGATGATGCGCG 900
CFT073_glpT      801 CTGCTGGGGATGGCCTGGTTCAATGACTGGCACGCGGCGCTCTATATGCCCGCTTTCTGCGCCATTCTGGTGGCACTGTTCGCCTTTGCGATGATGCGCG 900
O157Sakai_glpT   801 CTGCTGGGGATGGCCTGGTTCAATGACTGGCACGCGGCGCTCTATATGCCCGCTTTCTGCGCCATTCTGGTGGCATTATTCGCCTTTGCGATGATGCGCG 900

GU2019-E4_glpT   901 ATACCCCGCAATCCTGTGGCCTGCCGCCGATTGAAGAGTACAAAAATGATTATCCGGACGACTATAACGAAAAAGCGGAACAGGAGCTGACCGCGAAGCA 1000
CFT073_glpT      901 ATACCCCGCAATCCTGTGGCCTGCCGCCGATCGAAGAGTACAAAAATGATTATCCGGACGACTATAACGAAAAAGCGGAACAGGAGCTGACCGCGAAGCA 1000
O157Sakai_glpT   901 ATACCCCGCAATCCTGTGGCTTGCCGCCGATCGAAGAGTACAAAAATGATTATCCGGACGACTATAACGAAAAAGCGGAACAGGAGCTGACCGCGAAGCA 1000

GU2019-E4_glpT  1001 GATCTTCATGCAGTACGTGCTGCCGAACAAACTGCTGTGGTATATCGCCATCGCCAACGTGTTCGTTTATCTGCTGCGTTACGGCATCCTCGACTGGTCA 1100
CFT073_glpT     1001 GATCTTCATGCAGTACGTGCTGCCGAACAAACTGCTGTGGTACATCGCCATTGCCAACGTGTTCGTTTATCTGCTGCGTTACGGCATCCTCGACTGGTCA 1100
O157Sakai_glpT  1001 AATCTTCATGCAGTACGTGCTGCCGAACAAACTGTTGTGGTATATCGCCATCGCCAACGTGTTCGTTTATCTGCTGCGTTACGGCATCCTCGACTGGTCA 1100

GU2019-E4_glpT  1101 CCGACTTATCTGAAAGAGGTTAAGCATTTCGCGCTGGATAAATCCTCCTGGGCCTACTTTCTTTATGAATATGCAGGTATTCCGGGCACCCTGCTGTGCG 1200
CFT073_glpT     1101 CCGACTTATCTGAAAGAGGTTAAGCATTTCGCGCTGGATAAATCCTCCTGGGCCTACTTCCTTTATGAATATGCAGGTATTCCGGGCACCCTGCTGTGCG 1200
O157Sakai_glpT  1101 CCGACTTATCTGAAAGAGGTTAAGCATTTCGCGCTGGATAAATCCTCCTGGGCCTACTTCTTCTATGAGTATGCGGGTATTCCGGGCACCCTGCTGTGCG 1200

GU2019-E4_glpT  1201 GCTGGATGTCGGATAAAGTCTTTCGTGGTAACCGTGGGGCAACCGGCGTCTTCTTTATGACACTGGTGACCATCGCGACTATCGTTTACTGGATGAACCC 1300
CFT073_glpT     1201 GCTGGATGTCGGATAAAGTCTTTCGTGGTAACCGTGGGGCAACCGGCGTCTTCTTTATGACACTGGTGACCATCGCGACTATCGTTTACTGGATGAACCC 1300
O157Sakai_glpT  1201 GCTGGATGTCGGATAAAGTCTTCCGTGGCAACCGTGGGGCAACCGGCGTTTTCTTTATGACACTGGTGACCATCGCGACTATCGTTTACTGGATGAACCC 1300

GU2019-E4_glpT  1301 GGCAGGTAACCCAACCGTCGATATGATTTGTATGATTGTTATCGGCTTCCTGATCTATGGCCCTGTGATGCTGATCGGTCTACATGCGCTGGAACTGGCA 1400
CFT073_glpT     1301 GGCAGGTAACCCAACCGTCGATATGATTTGTATGATTGTTATCGGCTTCCTGATCTATGGCCCTGTGATGCTGATCGGTCTACATGCGCTGGAACTGGCA 1400
O157Sakai_glpT  1301 GGCAGGTAACCCAACCGTCGATATGATTTGTATGATTGTTATCGGCTTCCTGATCTACGGCCCTGTGATGCTGATCGGTCTGCATGCGCTGGAACTGGCA 1400

GU2019-E4_glpT  1401 CCGAAAAAAGCGGCAGGTACGGCAGCGGGTTTTACCGGGCTGTTTGGTTACCTGGGCGGTTCGGTGGCGGCGAGCGCGATTGTTGGCTACACCGTGGACT 1500
CFT073_glpT     1401 CCGAAAAAAGCGGCAGGTACGGCAGCGGGTTTTACCGGGCTGTTTGGTTACCTCGGCGGTTCGGTGGCGGCGAGCGCGATTGTTGGCTACACCGTGGACT 1500
O157Sakai_glpT  1401 CCGAAAAAAGCGGCAGGAACGGCAGCGGGCTTTACCGGTCTGTTTGGTTACCTGGGCGGTTCGGTGGCGGCGAGCGCGATTGTTGGCTACACCGTGGACT 1500

GU2019-E4_glpT  1501 TCTTCGGCTGGGATGGCGGCTTTATGGTAATGATTGGCGGCAGCATTCTGGCGGTTATCTTGTTGATTGTTGTGATGATTGGCGAAAAACGTCGCCATGA 1600
CFT073_glpT     1501 TCTTCGGCTGGGATGGCGGCTTTATGGTAATGATTGGCGGCAGCATTCTGGCGGTTATCTTGTTGATTGTTGTGATGATTGGCGAAAAACGTCGCCATGA 1600
O157Sakai_glpT  1501 TCTTCGGCTGGGATGGCGGCTTTATGGTAATGATTGGCGGCAGCATTCTAGCGGTTATCTTGTTGATTGTTGTGATGATTGGCGAAAAACGTCGCCATCA 1600

GU2019-E4_glpT  1601 ACAATTACTGCAAAAACGCAACGGAGGCTAA                                                                      1631
CFT073_glpT     1601 ACAATTACTGCAAAAACGCAACGGAGGCTAA                                                                      1631
O157Sakai_glpT  1601 GGAATTACTGCAAAAACGCAACGGAGGCTAA                                                                      1631
```
