# Supplementary material for: Inactivation of ackA and pta Genes Reduces GlpT Expression and Susceptibility to Fosfomycin in Escherichia coli
Source: Microbiol Spectr. 2023 May 18;11(3):e05069-22. doi: 10.1128/spectrum.05069-22 (PMC10269713; doi:10.1128/spectrum.05069-22)
Supplement: Supplemental file 4 — Supplemental material. Download spectrum.05069-22-s0004.pdf, PDF file, 0.2 MB [file spectrum.05069-22-s0004.pdf]

```
GU2019-E4_fis   1 CCTGGATCTTTCGGGAAATCCAGCATTATCTGGACACTGGGGAGTTGCTGCCCCCGCTGCCTTTGGCAGAGGTTAAGCGCTTGCTTTGCGCGCACGTTCG 100
CFT073_fis      1 CCTGGATCTTTCGGGAAATCCAGCATTATCTGGACACTGGGGAGTTGCTGCCCCCGCTGCCTTTGGCAGAGGTTAAGCGCTTGCTTTGCGCGCACGTTCG 100
O157Sakai_fis   1 CCTGGATCTTTCGGGAAATCCAGCATTATCTGGACACTGGGGAGTTGCTGCCCCCGCTGCCTTTGGCAGAGGTTAAGCGCTTGCTTTGCGCGCACGTTCG 100

GU2019-E4_fis 101 GGAACTGCATGACTTTTATGGTCCGGCAAAAGGGTACCGAATTGCACGTAAACACGTTTCCTGGTATCTCCAGGAACACGCTCCAAATGACCAGTTTCGG 200
CFT073_fis    101 GGAACTGCATGACTTTTATGGTCCGGCAAAAGGGTACCGAATTGCACGTAAACACGTTTCCTGGTATCTCCAGGAACACGCTCCAAATGACCAGTTTCGG 200
O157Sakai_fis 101 GGAACTGCATGACTTTTATGGTCCGGCAAAAGGGTACCGAATTGCACGTAAACACGTTTCCTGGTATCTCCAGGAACACGCTCCAAATGACCAGTTTCGG 200

GU2019-E4_fis 201 CGCACATTCAACGCCATTGAGGATGCCAGCGAACAGCTGGAGGCGTTGGAGGCATACTTCGAAAATTTTGCGTAAACAGAAATAAAGAGCTGACAGAACT 300
CFT073_fis    201 CGCACATTCAACGCCATTGAGGATGCCAGCGAACAGCTGGAGGCGTTGGAGGCATACTTCGAAAATTTTGCGTAAACAGAAATAAAGAGCTGACAGAACT 300
O157Sakai_fis 201 CGCACATTCAACGCCATTGAGGATGCCAGCGAACAGCTGGAGGCGTTGGAGGCATACTTCGAAAATTTTGCGTAAACAGAAATAAAGAGCTGACAGAACT 300

GU2019-E4_fis 301 ATGTTCGAACAACGCGTAAATTCTGACGTACTGACCGTTTCTACCGTTAACTCTCAGGATCAGGTAACCCAAAAACCCCTGCGTGACTCGGTTAAACAGG 400
CFT073_fis    301 ATGTTCGAACAACGCGTAAATTCTGACGTACTGACCGTTTCTACCGTTAACTCTCAGGATCAGGTAACCCAAAAACCCCTGCGTGACTCGGTTAAACAGG 400
O157Sakai_fis 301 ATGTTCGAACAACGCGTAAATTCTGACGTACTGACCGTTTCTACCGTTAACTCTCAGGATCAGGTAACCCAAAAACCCCTGCGTGACTCGGTTAAACAGG 400

GU2019-E4_fis 401 CACTGAAGAACTATTTTGCTCAACTGAATGGTCAGGATGTGAATGACCTCTATGAGCTGGTACTGGCTGAAGTAGAACAGCCCCTGTTGGACATGGTGAT 500
CFT073_fis    401 CACTGAAGAACTATTTTGCTCAACTGAATGGTCAGGATGTGAATGACCTCTATGAGCTGGTACTGGCTGAAGTAGAACAGCCCCTGTTGGACATGGTGAT 500
O157Sakai_fis 401 CACTGAAGAACTATTTTGCTCAACTGAATGGTCAGGATGTGAATGACCTCTATGAGCTGGTACTGGCTGAAGTAGAACAGCCCCTGTTGGACATGGTGAT 500

GU2019-E4_fis 501 GCAATACACCCGTGGTAACCAGACCCGTGCTGCTCTGATGATGGGCATCAACCGTGGTACGCTGCGTAAAAAATTGAAAAAATACGGCATGAACTAA    597
CFT073_fis    501 GCAATACACCCGTGGTAACCAGACCCGTGCTGCTCTGATGATGGGCATCAACCGTGGTACGCTGCGTAAAAAATTGAAAAAATACGGCATGAACTAA    597
O157Sakai_fis 501 GCAATACACCCGTGGTAACCAGACCCGTGCTGCGCTGATGATGGGCATCAACCGTGGTACGCTGCGTAAAAAATTGAAAAAATACGGCATGAACTAA    597
```
